# Self-Management in Older Pakistanis Living With Multimorbidity in East London

**DOI:** 10.1177/10497323211019355

**Published:** 2021-06-10

**Authors:** Najia Sultan, Deborah Swinglehurst

**Affiliations:** 1 Queen Mary University of London, London, United Kingdom

**Keywords:** United Kingdom, narrative methods, BNIM, self-management, multimorbidity, immigrants and migrants, qualitative

## Abstract

In this article, we explore how older British Pakistani people experience multimorbidity (defined as the coexistence of two or more medical conditions) and engage with self-management within the context of their life histories and relationships. We conducted biographical narrative interviews in Urdu and/or English with 15 first-generation Pakistani migrants living with multimorbidity, at their homes in East London. Our analysis showed that the triadic construct of family, faith, and health was central to how participants made sense of their lives, constituting notions of “managing” in the context of multimorbidity. For Pakistani patients, the lived experience of health was inseparable from a situated context of family and faith. Our findings have implications for existing public health strategies of self-management, underpinned by neoliberal discourses that focus on individual responsibility and agency. Health care provision needs to better integrate the importance of relationships between family, faith, and health when developing services for these patients.

## Introduction

It is both a great success and a significant challenge for global health care systems that people are living longer. The aging population is accompanied by a higher prevalence of long-term conditions (LTCs) and multimorbidity—defined as the coexistence of two or more medical conditions (Barnett et al., 2012). Multimorbidity places burden on affected individuals and the health care system and is associated with increased unplanned hospitalizations and impaired quality of life (Marengoni et al., 2011). Multimorbidity is more prevalent in certain groups including older adults and ethnic minorities (Schafer et al., 2012; Verest et al., 2019).

Self-management is the “lifetime task” of managing an LTC and is made more complex in the context of multimorbidity (Holman & Lorig, 2004). Corbin and Strauss (1988) described three groups of tasks that contribute to the self-management of LTCs: medical or behavioral management (e.g., taking medication, following a special diet), maintaining and creating new behaviors or life roles, and the emotional management of living with LTCs. Supporters of self-management claim that it empowers people to take control of their health (McCorkle et al., 2011). Interventions frequently focus on skills in problem-solving and goal setting (Newman et al., 2004) and are described by advocates as having broad cognitive, social, and psychological benefits (de Silva, 2011). Formal self-management programs such as the United Kingdom’s Expert Patient Program form part of a larger policy discourse directed at containing the escalating burden and cost of multimorbidity (Donaldson, 2003).

Critics identify self-management programs as constituting a “downward delegation” of work to patients, prioritizing theoretical cost-saving at the expense of holistic care (Rogers et al., 2009). They suggest that self-management programs are an extension of progressively neoliberal health care reform, occurring on a global scale and based on principles of individualism, privatization, and decentralization (McGregor, 2001). Critics further argue that framing self-management in morally emotive language of what is right and responsible distracts from these underlying neoliberal roots (Lawn et al., 2011).

It has been suggested that the self-management discourse underestimates the importance of the biographical precipitants of poor health and the socioeconomic and structural systems of inequality that create and sustain it (Dowrick, 2017). In practice, many formal self-management programs have been shown to have limited, poorly sustained success despite the “religiosity” with which these programs are advocated by policy makers (Rogers et al., 2009).

There are particular concerns about the effectiveness of self-management programs among ethnic minority groups that are known to carry a disproportionate burden of LTCs (Vyas et al., 2003). Scholars note that cultural context is of vital importance when seeking to understand how individuals self-manage LTCs; culture shapes how individuals make sense of symptoms as well as why and how an individual may utilize their resources to seek care (Helman, 2007). Critics have raised concerns that existing self-management programs often pay little attention to the social factors shaping the lives of the intended recipients and, instead, the programs themselves become a source of inner conflict and stress for patients (Audulv et al., 2012; McDonald et al., 2016).

Increasingly, self-management research suggests that much of the “work” of living with LTCs is collaborative (Polak & Green, 2020). Existing research, typically focused on English-speaking patients with specific diseases, highlights the importance of family networks in managing the burdens of LTCs (Boise et al., 1996). Studies in Hispanic and African American populations have furthermore also identified a stronger role for religious beliefs in making sense of chronic illness compared with White populations (Juarez et al., 1998; Koffman et al., 2008).

### Self-Management in the British Pakistani Community

Britain is home to 1.17 million Pakistanis, the largest Pakistani community in Europe (Office for National Statistics, 2016). Pakistani migrants remain relatively disadvantaged with an excess and premature toll of LTCs compared with other ethnic groups in Britain (Evandrou et al., 2016).

The mass migration of Pakistanis to Britain started in the 1950s following the 1948 British Nationality Act, conferring British citizenship on all Commonwealth subjects and recognizing their right to work and settle in the United Kingdom (Ansari, 2013). Many were employed in factories with tough working conditions and frequent exposure to discrimination. These migrants were frequently from the poorer, working classes and spoke Punjabi as a first language. Although Urdu is spoken as a first language primarily by Pakistan’s wealthy classes, it is widely spoken throughout Pakistan as the official national language (Rahman & Knight, 1996). A total of 97% of Pakistanis identify as Muslims and for most first-generation Pakistanis, being Pakistani and being Muslim are synonymous identities (Jacobson, 1997).

South Asians comprise one third of the population of Tower Hamlets and Newham, the boroughs in which we conducted this research. These are two of the United Kingdom’s most ethnically diverse and deprived local authority areas (Ministry of Housing, Communities & Local Government, 2019). Previous research has shown that multimorbidity occurs 10 to 15 years earlier in the most deprived areas in the United Kingdom, with significantly higher rates of physical–mental comorbidities (Cassell et al., 2018). There has been considerable focus on the role of social determinants and “health literacy” in contributing to the poor health of many ethnic minorities groups, but it is well recognized that these factors alone, while important, do not adequately explain persistent disparities.

Current literature on self-management in the Pakistani population is mostly disease-specific and focuses predominantly on diabetes. Lawton et al. (2008) conducted interviews with British Indian and Pakistani type 2 diabetics, exploring dietary habits. Participants maintained that, although they felt a responsibility toward their health, their commitment to families and communities meant it was important for them to enjoy social gatherings, even if this compromised health outcomes. Thus, knowledge of “healthy” eating was neither necessary nor sufficient to support healthy eating (as conventionally understood), and understandings of what it meant to be “healthy” needed to be understood within a wider social context.

In a separate study of accounts of diabetes causation, Lawton et al. (2007) found that White respondents expressed the rhetoric of Western personhood and described their diabetes onset in terms of self-blame and individual choices. Conversely, Pakistani participants demonstrated a “socio-centric sense of self,” externalizing responsibility for their disease, describing a life determined by their circumstances, and the will of God in ultimately “dictating health and destiny.” Lawton concludes that “a line of questioning which implicated the self in the onset of the disease . . . may have been ethnocentric” (pp. 891–906).

A study conducted by Mir and Sheikh (2010) indicated that Pakistani Muslim patients valued practitioners making reference to their faith as a therapeutic resource and a means of facilitating emotional adjustment to LTCs. Their analysis also revealed a dichotomy “between the significant personal resource that faith provided and the discrimination that Muslim identity triggers in UK society.” During health care encounters “Pakistani patients (would make) this aspect of their identity almost invisible, with adverse consequences for treatment” (p. 337).

## Method

This was a narrative interview study. The aims were to explore how older Pakistani adults experience multimorbidity and to illuminate how they “manage” their selves and their health in the context of their daily lives.

This research is novel in two ways. First, we used the Biographic Narrative Interpretive Method, an open-ended storied approach, which invited participants to respond to a single question in whatever way, and for as long as they wished (Wengraf, 2001). This “unstructured” approach has not been used previously among older British Pakistanis to explore multimorbidity. Participants identified which aspects of their health and illness experience held value to them personally and narrated this experience *in their own words and in their own language*.

Second, although a body of research has shown the value of exploring the experiences of ethnic minority patients with single diseases, our study elicited narratives in which participants articulated how they integrated their *multiple* chronic conditions into their lives. This is particularly important, given that multimorbidity may amplify the burden and complexity of the work involved in “managing.”

### Sampling

A total of 15 people were recruited through seven general practitioner (GP) practices in East London. The inclusion criteria were as follows: people aged more than 50 years (to reflect the early onset of multimorbidity in this population) of Pakistani ethnicity who spoke Urdu or were bilingual Urdu–English speakers, with two or more ongoing medical conditions. A GP in each practice conducted an electronic search to identify eligible participants, sent invitation packs in Urdu and English, and sought verbal consent from interested participants for a researcher to make telephone contact to explain the study. Sampling was purposive to identify maximum diversity across the following attributes: age (53–87 years), gender (nine females, six males), educational background (two received no formal education, 11 completed education between ages 12–18 years, and two completed degrees), and comorbidities. Participants had, on average, four comorbid conditions and, across the sample, there were 21 comorbid conditions. Hypertension (15 people) and diabetes (13 people) were the most common conditions, but experiences included heart failure, hypothyroidism, depression, epilepsy, asthma, and osteoarthritis, for example. Three participants self-identified as fluent in conversational English and 12 participants described themselves as speaking little or no English. All were resident permanently in the United Kingdom (average 33 years). A total of 13 participants lived with adult children and one participant had a formal carer. Participants were prescribed an average of 12.6 regular medications.

### Data Collection

Fifteen in-depth narrative interviews were conducted in participants’ homes, 13 in Urdu and two in English. In comparison with academic or clinical settings, the home is a more informal environment for a biographical interview and the location of most of the “work” of multimorbidity (Corbin & Strauss, 1985). Participants were invited to have someone present with them at the interview if they wished; six chose to have their family present. Written informed consent forms were available in Urdu and English and were signed by all participants. The interviewer was an Urdu-speaking Pakistani female and a doctor working in the GP setting. She introduced herself as a university researcher. None of the research participants were recruited from the GP practice in which she works.

Interviews were conducted using Wengraf’s (2001) Biographical Narrative Interpretive Method (BNIM). BNIM interviews begin with a “single question aimed at inducing narrative” (SQUIN). The SQUIN was as follows: *Can you please tell me your story of your life since you were first advised to take medicines. I would like you to tell me about all the experiences, people, and events which are important to you personally. Please begin wherever you like, and take as long as you need.* The participant responded to the SQUIN uninterrupted by the researcher, who noted cue phrases on which to build the interview. After a short break, the interview continued with the researcher selecting a range of cue phrases, in the order in which they were introduced within the SQUIN to elicit further, more detailed narratives. This was followed by a short semi-structured interview, based on a topic guide to fill gaps. The topic guide included questions regarding the routines and practical arrangements for taking medication and medication reviews, and questions regarding what worked well and what were the challenges of living with multimorbidity. Interviews took place between December 2017 and June 2018 and lasted an average of 72 minutes. Interviews were audio-recorded, transcribed, and translated into English. Transcripts were anonymized prior to analysis.

### Data Analysis

We followed Muller’s (1999) four steps of narrative data analysis: data entry (reading, sorting to gain familiarity), sensemaking (finding connections, patterns through successive readings, and reflection), verifying (searching for alternative explanations, confirmatory and disconfirming data), and accounting for what has been learnt. Transcripts were initially coded manually and then on NVivo where coding categories and themes were organized and discussed between the authors. Data workshops involving members of the multidisciplinary Apollo-MM research team were used to develop and refine our analysis. A reflexive research journal was kept throughout.

The study was granted a favorable ethical opinion by North East Tyne & Wear South Research Ethics Committee (IRAS project ID: 228870; REC reference 17/NE/0314) through proportionate review.

## Findings

Participants experienced, understood, and articulated “multimorbidity” (although unsurprisingly none used this term) with reference to family, faith, and health. They understood their health to be affected by—and in turn, to affect—their relationship with family and with God, in a deeply connected recursive triadic interrelationship. The relationship between family and health was accounted for in two ways. First, emotional family events, particularly deaths and losses, were frequently identified as being a *cause* of personal ill-health. Second, family was seen as a source of emotional and practical support in *managing* ill-health. Furthermore, all but one participant identified the importance of their Muslim faith in making sense of, and managing their ill-health. Beyond the central triad of family, faith, and health lay a wider circle of concern, comprising the participants’ community. Our analysis further illustrates how participants presented themselves to this wider community as good patients and citizens. In the following, we discuss our findings and interpretations, linking them to the wider literature within the three themes identified above.

### Critical Events in Families as a Cause of Ill-Health

Throughout the interviews, accounts frequently attributed the onset of chronic ill-health to stressful family events. The participant, in the following narrative, was interviewed with her husband present and describes her experience of emotional distress and resultant physical symptoms she attributed to marital problems. Their multigenerational home included the children of her husband’s second marriage, which was conducted under Islamic law in Pakistan (where more than one wife is permitted) and was initially kept secret from the participant. Despite fertility treatment, the participant did not herself have children. In this extract, her reference to “getting sick” relates to the onset of valvular heart failure, which she attributes to the sorrows that “settle themselves” in her heart:

What happens is, you get ill because there are certain sorrows in life that settle themselves in your heart. And from that, your illnesses keep growing, from thinking about it . . . I couldn’t have children so he [my husband] got married again. That caused me a great deal of sadness . . . That almost killed me—the sadness . . . And this second marriage, remarrying—that’s when I started getting sick and all my illnesses got much worse . . . The thing is nobody knows these things about you. It’s only the person suffering who knows what they are going through.

Within Islamic interpretations of health, the body and mind are understood to be interconnected and Pakistani patients frequently present to doctors with physical symptoms which they themselves attribute to psychological or emotional distress (Mumford et al., 1991). Within Western ideology and health systems, there is often a tendency for biological explanations of ill-health to trump biographical explanations (Heath, 1995). Patients, such as many in this study, with somatic presentations of emotional distress may create diagnostic confusion for practitioners and become exposed to unnecessary investigations and treatments. Although not unique to patients of Muslim background (Biderman et al., 2003), these patients may be vulnerable to the consequences of misunderstandings arising from different cultural constructions of symptoms.

In our interviews, narratives involving death or loss of a loved one were commonly situated in accounts of new diagnoses. One participant, who had migrated to London aged 16 years, recalls his new diagnosis of diabetes in the following extract. Although this account was in Urdu, he twice used the word “shocking” in English, placing particular emphasis on the unexpected nature of his father’s death and juxtaposing the account with his sense of geographic separation from his father in Pakistan:

In 1997 my father died and that was shocking for me. He was in Pakistan—it was very shocking. My younger brother and I were here and so we went to Pakistan for the funeral. After that I told the doctor about my pains. He told me to give a sample of urine for a test. He told me I had diabetes and told me there was no medicine yet . . . . After six months he gave me a tablet . . . . it started from there and MashAllah (Arabic, As God has willed) now I am on 22 medicines including insulin.

This participant constructs the temporal, causative interdependence (“after that”) between emotional distress and resulting somatic illness (“my pains”), which in turn becomes a diagnosis of diabetes. “Biographical disruption” was a term coined by British sociologist Michael Bury to describe the process where critical situations in life become a basis for a change in identity. According to Bury (1982), “chronic illness, is precisely that kind of experience where the structures of everyday life and the forms of knowledge which underpin them are disrupted” (p. 169).

Female participants in our study often attributed their ill-health to difficult marriages. Marital difficulties as a source of mood disorders among Pakistani women are well documented (Chaudhry et al., 2012). In the following interview, the narrator consistently attributed a physically abusive marriage, when aged 16 years to her first cousin, as the source of her ongoing ill-health. She recounted experiences of numerous abuses, including being strangled and poisoned. Close-knit family networks made separation difficult, but she eventually escaped to live with extended family in London where she claimed asylum. This participant was tearful while giving this account, expressing visible guilt at how her actions to protect her daughter and herself came at the expense of leaving her sons back in Pakistan with her own mother. Earlier in the interview, she described her distress when her husband refused to pay for an inexpensive medicine for her son (“Even now when I think about it my hairs stand on end”) and spoke of her high regard for easy access to medical treatment in the United Kingdom:

You cannot think how I got out of Pakistan . . . how I got here. I left two children behind. My son was five years old when I came here. I didn’t even meet him before coming. What kind of mum leaves her children? . . . Then [my children] didn’t get the visa [to join me in the UK] . . . . You know when your kids are separated, what it does to a mother’s kaleja (Urdu, Liver^*^) It is broken into two pieces—one in Pakistan and one here. I can’t leave here and go to Pakistan . . . . In Pakistan I have no way of making any income. First thing in Pakistan is I don’t have money for any medical treatment.

^*^“Kaleja” in Urdu refers to the liver, although it is metaphorically equivalent to the Western notion of the heart as a seat of emotion.

She goes on to describe her recurring panic attacks and her ongoing reliance on sleeping tablets, the need for which is attributed to the distressing experiences she has lived through:

They are giving me sleeping medicines, and I am taking those because at least at night I can sleep in peace. I see things . . . . I hear a child crying mum . . . sometimes I feel like someone is trying to strangle me.

There is increasing evidence of the association between traumatic life events, including structural violence, marital issues, and racism with the development of chronic illness (Renzaho et al., 2013). According to Kirkengen (2010), treating chronic illnesses in such cases with biomedical approaches alone may perpetuate adversity by failing to help people make sense of and integrate these experiences into their lives: “When we suffer health problems due to violations of our embodied selves, a model that ignores subjectivity and fails to link body with selfhood is a failure” (p. 398). Rudebeck (2000) identified “bodily empathy” as a core skill of general practice, suggesting that a doctor must master recognizing a patient’s subjective experience before the patient is able to trust that doctor’s interpretation of their illness.

In their studies of stroke survivors in East London, Pound et al. (1998) documented the extent to which participants downplayed the effect of stroke on their lives. The authors note that

the older members of this group had experienced war, poverty and loss, while some of the younger members were migrants to the East End and were already dealing with serious problems, including unemployment and homelessness. For most people then, the stroke entered a life already characterised by struggle and hardship. (p. 498)

We encountered a similar embeddedness of illness narratives within stories of hardship, past, present, and ongoing. We end this section with some notes written by the interviewer in a reflexive journal (Box 1):

**Table:** 

As a member of the Pakistani community in London I reflect on the stories of hardship that I keep on encountering in my interviews and the ways in which interviewees position themselves within their stories. I sense that displaying an excessive concern for ones’ self and ones’ personal problems might be construed as self-involved within a collectivist society such as ours and wonder how this is shaping the accounts I am hearing. At the same time I am struck by how a display of concern and sadness for ones’ family would be regarded as noble and “right” and how participants might try to balance these concerns in how they present themselves to me. Most of the interviewees address me as “beta” (meaning daughter), similar to what happens in my clinical role. Whilst this is culturally appropriate given my younger age, this perhaps also suggests that my interviewees are conscious to project themselves as good and moral members of the community to a younger Muslim Pakistani like me, who—in turn—would be expected to look upon wise elders, like them, as an example. Extract from reflexive journal (June 5, 2018)

### Box 1. Family Networks in Managing the Work of Multimorbidity

Older adults with multimorbidity are frequently reliant on the support of family caregivers, and a lack of family support has been identified as a barrier to engaging with self-management routines (Minet et al., 2011; Ploeg et al., 2019). Our interviewees consistently recognized the important role of their family networks in managing multimorbidity in accounts that resonate with May et al.’s (2014) observations that capacity to engage with self-management is not dependent on an individual’s performance alone, but also reliant on their relational networks social skill, social capital, and socioeconomic resources.

In our study, it was usually adult children who performed advocacy roles as spouses often did not speak English. Parents frequently described their adult children as culturally aware facilitators in navigating the health care system and seeking help when needed. Some drew particular attention to how “good” their own children were compared with “other people’s children.” Having to balance the requests of parents within the “rules” of seeking medical help was complex, as well illustrated in accounts of this female participant whose escalating tramadol use for arthritis (a highly addictive painkiller) was a source of conflict at home. She describes being started on tramadol by doctors but seems frustrated by the “many questions” she is asked by doctors for ongoing prescriptions:

At the beginning the doctor . . . he was the one who started me on these tablets. I said to him I don’t take it . . . . now without the medicine I am not at peace . . . whenever I go they ask me so many questions . . . they say your mum she takes too much tramadol . . . she does this, she does that.

Note that questions from doctors about her care are posed to her son (“they say your mum”). Her son went onto further elaborate to the interviewer, the practical and emotional demands of obtaining this tightly controlled medication from his mother’s GP on a regular basis, and the considerable stress this placed upon the wider family unit.

Reliance on children can additionally be problematic from the perspectives of the parents. Our findings include accounts when distributed care within families may have fallen short of prioritizing the patient’s best interests. In the following narrative, a different participant describes the significant role of their children in deciding whether their father went for back surgery. There is frustration from the participant that their children’s’ involvement in decision-making resulted in a canceled operation and the ongoing problem of recurrent falls arising from this:

They told us [my husband] needs to go for an operation . . . [and] that if he had the operation he could die . . . or he could be paralysed. My son went, my daughter, my daughter-in-law, all of them went to the next appointment and told the doctors that he couldn’t have the operation . . . . But now he [my husband] can’t get out of the house. He has fallen over quite a few times . . . . So now the children don’t let us go out . . . But they were the ones who had the operation cancelled.

Of note, the voices of the participant and her elderly husband are erased in this account of the decision-making process and they position their children as continuing to contribute to their ongoing difficulties because “they don’t let us out.”

In Stenberg and Furness’s (2017) interview study of people with LTCs, participants described a sense of isolation that accompanied chronic ill-health. The authors noted that for their participants, social connectedness was a key component in “living well” with LTCs. In our study we noted that, in addition to family involvement, there were several stories of practical and emotional support from friends within the local cultural community in East London. In comparison with stories of reliance on children, participants frequently described friendship networks as providing more mutual support. One participant, now aged 72 years, had migrated to the United Kingdom as an older adult to join her economically successful children. For years she had been meeting a group of older Pakistani women in the same park, at the same bench, every weekday for morning walks, exemplifying a locally fostered, informal social network: “When us friends go for a walk, we stand and chat about our pains and problems.” This network clearly provided her a valuable sense of support with peers with whom she shares a history, language, and beliefs.

Our findings consistently demonstrate that, for this older Pakistani population with multimorbidity, the emotional and practical work of living with multimorbidity was underpinned by the support of their networks; most frequently consisting of the immediate family. General sociocultural expectations within this community dictate that those at the extremes of age, namely, young children and older people, are the responsibility of those deemed to be at the peak of their mental and physical capabilities. Our interviews suggest that adult children often had a significant say in medical decisions regarding their parents as they advanced in age, even in situations in which their parents retained mental capacity to make autonomous choices. Thus, although the nature of the reciprocal relationship of the parent and “child” changed significantly through the life span, the shared moral commitment to this reciprocity as a fundamental expression of kinship remained. As one participant reflected, “I consult my son to ask him what I should do. Before I would advise my family for everything, now I consult them.” The consistent support of family networks juxtaposes with the feeling of deterioration participants often describe, from that of being an active independent healthy person to that of more dependent patients. Ho (2008) noted in her article on medical decision-making, that

family members, who are the constants in a changing plethora of health professionals and whose relations with the patient have been a part of the individual’s identity, are reminders that the patient is not a mere collection of dysfunctional body parts that require professional intervention, but a moral agent. (p. 131)

Our analysis shows that for the older Pakistani people in this study, notions of self and agency are deeply entangled with kinship. Although largely supportive, there are contrasting examples, in our interviews, of social networks and familial support further complicating how individuals self-manage their LTCs. Nonetheless, the predominance of narratives about kinship in our findings is seemingly at odds with much of existing health policy, centered on individual choices and autonomous self-management.

### Faith and Health

The importance of spirituality in the context of LTCs has been demonstrated in many populations. Friedemann et al. (2002) suggested that spirituality takes on special relevance when change is irreversible, such as chronic illness is irreversible. In our study, the interviewer was headscarf-wearing and visibly Muslim, which may have enabled participants to discuss their faith more openly. The use of common Islamic expressions (e.g., Alhumdullilah [Arabic, *Praise be to God*], MashaAllah [Arabic, *As God has willed*]) throughout interviews reflected a shared understanding and attributed “insider status” to the interviewer as a member of the Muslim community. Arguably, while enabling a particular kind of access to this community, this may also have prompted talk deemed “appropriate” between Muslims.

Many participants referred to Islamic beliefs to explain how they made sense of and coped with deteriorating health. This included being grateful for all things—even ill-health—in a frame which positioned both health and ill-health as God’s will and, by implication, to be accepted stoically: “*Even if I get unwell, well, illness is from God.*” The following quote from one participant incorporates several recurring subthemes we encountered regarding faith and health: the importance of forgiveness, gratitude, and having faith in God’s plan.

All I ask is that God forgives me. There is nothing in my brain or my heart—just all I think is Alhumdullilah. But these worldly matters—they will carry on. They will only end when—Inna Lillahi Wa Inna Lil Rajioon (Quranic Arabic (2:156): Indeed, to God we belong and to God we shall return). They don’t end. They will carry on. I just thank God. That he gives illness because even illnesses come from him. You just keep praying and he keeps listening. Just think—if you have something of your own—would you ever ruin it? No. We are all his. We need to try and he will make it better for us.

Coventry et al. (2015) proposed that for many people experiencing chronic ill-health, “multimorbidity is less about a collection of illnesses but is often experienced as a complex state oscillating between existing (getting through the day) and nonexistence (running down time on a life)” (p. 6). Many Muslims see reflecting frequently on death as a way of being consistently mindful of God, and existing research supports the notion that religiosity is associated with decreased anxiety about death (Daaleman & Dobbs, 2009). We found our participants frequently spoke about death and the afterlife in interview, with one participant reflecting, “*We all have to leave [this world]—He just hasn’t told us which time.*” A belief in the afterlife is common among believers of many faiths. A study of Black Caribbean cancer patients similarly identified the concept of moving onto a “better life” after death, one which was “a richer existence than their mortal presence on earth” (Koffman et al., 2008, p. 786).

Hawking et al. (2020) noted during narrative interviews with people on oral anticoagulation medication that when discussing medication taking, patients undertook significant discursive work to construct themselves as “good” patients. This was evident in our narratives where being a “good” patient was seen as an extension of gratitude to care received. Gratitude is central to Islamic faith and the Islamic tradition holds medicine and medical practitioners in particular high regard; the Quran (5:32) states that “if anyone saved a life it would be as if he saved the life of the whole humanity” (Haleem, 2010). Our interviews included frequent narratives of gratitude for access to high-quality and “free” health care available through the National Health Service (NHS): “*Here, thank God, medicine is free, doctors are free, hospitals are free.*” These narratives of gratitude were often juxtaposed with accounts of very poor access to health services in Pakistan where participants regarded care to be of poor quality and prohibitively expensive: “*Back home, if you become ill you die because you can’t get the treatment.*”

Despite gratitude for the NHS and the care provided, we encountered several accounts of participants undertaking self-management practices that they concealed from their Western health care providers, so that participants could fulfill their religious obligations. Although fasting during Ramadan is an important part of the Islamic faith, it is widely recognized that older and sick believers are exempt from fasting. Existing research suggests, however, that many Muslim patients with LTCs will fast regardless. In one interview study of British Muslims’ attitudes to fasting, some respondents described both avoiding health services during Ramadan and making adaptions to their medication regimens without their doctor’s knowledge to allow them to fast (Patel et al., 2015). We encountered similar practices in our study even among participants with very complex medical needs and medicine regimens. One participant who had asthma and several other medical conditions, describes making adaptations that extended to taking steroid medications obtained from abroad without medical advice, so that he can “self-manage” his asthma during Ramadan, allowing him to fast.

It is for asthma, when you have an attack they give you a course of it . . . . the doctor does not give it, he doesn’t know either and I don’t tell him . . . Sometimes I get it from Saudi Arabia and I save it, it lasts two or three years. From that my breathing doesn’t go wrong . . . First Ramadan I took it, I realised I cannot live without it, it’s a good one . . . . It is also a mental thing, where if I take this I feel I will be okay. If you have prayed to God to help you and then you are also taking this tablet (Laughs) . . . . It is not that if you just pray you will be fine. No. You should take medicines too. Do both.

Although he acknowledges that what he is doing would not have his doctor’s approval, he feels that combined with prayer and faith, he will be “*okay.*” He does not appear concerned that his actions may be harmful and presents an account in which medicines are a force for “good” (“*I cannot live without it*”). He thus aligns his individual understanding of medical treatments with his individual needs from the perspective of his faith, bypassing conventional biomedical advice through his regular doctor.

Muslim scholars acknowledge the “wholeness of being” in which health involves spiritual, psychological, physical, and moral components. The complex, and possibly risky, arrangements that this participant undertakes to contribute to his spiritual health and “wholeness” demonstrate this orientation. Individualized self-management strategies that place surveillance and management of the physical “self” at the center are frequently not consistent with Islamic values and practices in which God is regarded as the mediator of benefit in all things, and health holds meaning beyond the physical. Such individualized strategies may have limited success in this population.

With reference to faith and health, our interviews highlighted the centrality of faith to our participants’ modes of sense-making and action in the context of their multimorbidity. In particular, their accounts highlight the extent to which faith practices and forms of knowledge that align with faith are prioritized over “conventional” biomedical explanations which typically lie at the center of self-management efforts within existing Western health care provision.

## Discussion

This study explores how older adults from Pakistani backgrounds experience multimorbidity. Our overarching finding is that Pakistani patients make sense of their LTCs in a highly relational way, tied strongly to notions of family and faith. With 1.7 billion Muslims globally, our findings have relevance beyond East London, especially in situations where health care is informed by Western, predominantly secular ideologies. Our research suggests that the logics of “self management” within contemporary chronic disease management poorly reflect how these people understand and manage living with multimorbidity, especially by limiting its scope to the individual body and patients’ efforts to monitor and manage their singular selves.

This is, to our knowledge, the first study that explores multimorbidity in the British Pakistani population by adopting the BNIM. The researcher’s professional role within general practice opened avenues for recruitment within local primary care networks from a population known to be hard to reach (Nolde & Smillie, 1987). There were interviews where participants described going against the advice of their clinical teams. In accordance with our ethics approval, these reports were discussed between the researchers (both of whom are GPs), with a view to drawing this to the attention of the participant’s responsible clinician if we were concerned there was significant risk of harm. No such occasions were encountered. As an “insider” within the British Pakistani community, the interviewer shared language and cultural understandings that fostered an open discussion of sensitive topics such as death and hardship, building a data set rich in detail and emotion. Arguably, this “insider” status may have strongly shaped our participants’ narrative accounts, giving prominence to some story lines over others.

We join others in our critique of the predominance of the biomedical model as a means of understanding and caring for people with chronic disease. Scholars note that, within clinical encounters, too often “biological approaches trump biographical interpretations of patient’s problems, rendering the latter invisible or their relevance inconceivable, leading to harmful over-diagnosis and medicalization of human suffering” (Hjörleifsson & Lea, 2017, p. 28). Our biographical approach shows that many people assign clear associations between lived experiences of hardship and the onset of their chronic ill-health. These participants’ mindfulness of death may, furthermore, also be at odds with the aspirations of chronic disease management and its focus on delaying death. Although our study focused on narratives rather than clinical interactions, our findings lend support to the notion that clinical consultations may well be more effective if opportunities are provided for patients to explain how they understand their illness and where their hopes and fears lie. Among our participants, hope was only partially invested in biomedical solutions and, although doctors were held in high regard, fear that doctors may not accommodate faith commitments and wider biographical concerns led some to take action that may have been potentially harmful. This was suggested in our findings, and evidenced in previous studies, where patients described withholding information from their medical practitioners about fasting in the month of Ramadan, for fear of being told not to (Patel et al., 2015).

For the participants in this study, management strategies centered on personal responsibility are widely detached from how they make sense of, experience, and seek to manage their own ill-health. Western bioethics identifies respect for individual autonomy as one of the four prima facie principles. Consistent with wider literature of clinical decision-making, however, our participants’ decisions appear to be highly distributed within family networks, with the active involvement of family often appearing to be integral to their own sense of agency (Rapley, 2008). These findings strengthen existing arguments that a more relational concept of autonomy within health care has potential in improving the coherence of patient-centered care for many populations.

Our study, furthermore, casts some doubt on whether “self” management, as it is usually intended in health policy, can achieve useful ends within a population where the self is fundamentally social, deeply interconnected with wider social networks, and committed to a faith that holds that God’s will is the ultimate influence on long-term outcomes. Importantly, our participants did not frame God’s will, nor the role of their family and social networks, *in opposition to* medical intervention. Indeed, one said, “I ask God for my medicines and my doctors deliver them to me.” But the closely entangled “triad” of family, faith, and health as a “network of value” is a construct that we believe clinicians should attend to if they seek a shared understanding of patients’ lives as a basis for prudent decision-making.

Crawford (1980) coined the term “healthism” in 1980 to describe “the preoccupation with personal health as a primary focus for the definition and achievement of well-being; a goal which is to be attained primarily through the modification of lifestyles” (p. 368). Self-management programs and “healthism” are expressions of wider neoliberal constructs in which good health rewards individuals who take responsibility and adopt a proactive approach to health. Within “healthism,” lifestyle changes such as healthy eating and exercise are predominantly regarded as entirely personal choices. However, the “choice” to prioritize one’s individual health is challenging for people whose priority setting is necessarily entangled with close-knit family and social networks who collectively also share important religious obligations.

Scholars have suggested that the preoccupation of health care professionals, with biomedicine and personal autonomy, risks disregarding networks that patients most value and that this may leave patients who are managing LTCs to feel confused and conflicted (Ho, 2008). Our findings would support the idea that for certain populations and patients, family-based approaches to chronic disease management would be most appropriate. Certainly, if social networks contribute importantly to a patient’s sense of “self” and the constitution of agency, as our study suggests, then it is possible that conventional approaches to care, which focus so sharply on an individualistic notion of “selfhood,” may inadvertently perpetuate inequalities thereby affecting people already disadvantaged by older age or ethnic backgrounds. Furthermore, systems that incentivise standardized population-wide approaches to chronic disease management in the service of quality of care and equity may—in the context of diverse multiethnic populations—be focusing on what “good” care *looks like*, at the expense of what “good” care actually *is* for some patients. As Keller (1997) noted,

autonomy has been thought of as the pinnacle of human achievement . . . yet the capacity to form and maintain relationships, which has received little attention in the Western philosophical tradition, is arguably just as much of an achievement as autonomy, and just as important for moral maturity. Autonomy is one human good, and the ability to make and sustain connections with others is another; both are necessary for a full and rich human life. (p. 154)

## Conclusion

Our research highlights a disconnect between “self management” as it is formalized in policy, protocols, and practice and the narratives told by older British Pakistani people who are living with and managing multiple LTCs. For our older Pakistani participants, the lived experience of health was frequently inseparable from a situated context of family and faith. The triadic construct of family, faith, and health is central to how they make sense of their lives and how they “manage” in the context of multimorbidity. Current policy and practice regarding “self management” offers a version of responsible individual self-care that does not resonate with the understandings and needs of this group. Such approaches may be both impractical and unrealistic for many and risk exacerbating systemic disadvantage.
